# Placental biomarkers for the prediction of neurodevelopmental disorders

**DOI:** 10.3389/fcell.2025.1663960

**Published:** 2025-10-07

**Authors:** Payal Patel, Joy Ku, Ike Uzoaru, Jeffery A. Goldstein

**Affiliations:** ^1^ Carle Illinois College of Medicine, Urbana, IL, United States; ^2^ Department of Pathology, Carle Foundation Hospital, Urbana, IL, United States; ^3^ Department of Pathology, Northwestern University Feinberg School of Medicine and Northwestern Memorial Hospital, Chicago, IL, United States

**Keywords:** placental biomarkers, neuroplacentology, proteomics, trisomies, hypoxic-ischemic encephalopathy

## Abstract

Neurodevelopment shapes how children think, move, and engage with their surroundings. Understanding the pathways underlying neurodevelopmental pathophysiology in the perinatal stage can inform intervention strategies to mitigate or reduce the severity and extent of developmental brain injuries. Early risk stratification enables timely therapies and resource planning during a critical period for the developing brain. Over the past decade, attention has turned to the placenta as a uniquely informative vantage point for the identification of pregnancies at high risk for adverse neurodevelopmental outcomes. Situated at the maternal-fetal interface, the placenta functions as a dynamic record of intrauterine conditions, integrating genetic and environmental signals into distinct and quantifiable biomarkers. Emerging evidence indicates these placental biomarkers may predict later neurodevelopmental outcomes, highlighting the organ’s value in precision perinatal care. With this in mind, the objective of this scoping review will be to investigate the current use of placental biomarkers as predictors of neurodevelopmental outcomes in clinical practice, particularly the trisomies (T13, T18, T21). In the second section of this paper, we will focus on recent advancements and improvements in the use of placental biomarkers for diagnostic and prognostic purposes in other neurodevelopmental outcomes. Finally, this article concludes with a discussion of the impact of neuroplacentology in protocol development, risk stratification, and psychosocial wellness of pregnant women.

## 1 Background

As the first fetal organ to develop, the placenta defines the intrauterine environment and regulates maternal-fetal communication through nutrient exchange, immune modulation and endocrine signaling (Burton and Fowden, 2015). The Developmental Origins of Health and Disease (DOHaD) theory emphasizes the pivotal role of the placenta in shaping long-term outcomes for childhood health development (Ganguly et al., 2020).

Within this framework, neuroplacentology is an emerging field focused on exploring how placental structure and function influence fetal and neonatal neurodevelopment (Kratimenos et al., 2019). Abnormal placental signaling due to hypoxia, inflammation, or disrupted hormone regulation has been implicated in perinatal brain injuries and is associated with increased risk of neurodevelopmental disorders (Gall et al., 2022; Espinoza et al., 2022; Zhou et al., 2023; Vacher et al., 2021).

Given this connection, perinatal biomarkers have been established for the testing of fetal diseases. In one example, the American College of Medical Genetics and Genomics (ACMG) recommends the use of noninvasive prenatal testing (NIPT) for fetal chromosome abnormalities (Trisomy 13, 18, and 21) (Page et al., 2017). NIPT utilizes cell free fetal DNA (cffDNA) extracted from maternal plasma, which undergoes whole genome sequencing to screen for fetal aneuploidy (Nelissen et al., 2011).

In neuroplacentology, researchers are studying epigenetic markers and autoantibodies to develop minimally invasive liquid biopsy methods to determine a fetus’s risk for neurodevelopmental conditions.

Within the scope of this review, we will look at specific types of biomarkers that are used for the prediction and prognosis of neurodevelopmental outcomes. While the field also focuses on physiological factors that affect fetal development, such as maternal preeclampsia, our discussion centers around the communication systems themselves and how they may induce pathological developmental patterns.

## 2 Methods

We conducted a scoping review in accordance with the Preferred Reporting Items for Systematic Reviews and Meta-Analyses for Scoping Reviews (PRISMA-ScR) reporting guidelines. To identify potentially relevant documents, the following bibliographic databases were searched from April 1990 to 14 March 2025: PubMed and Scopus. The final search strategy can be found in Supplementary Table 1, and the list of papers included within the final review found in Supplementary Data Sheet 1.

### 2.1 Eligibility criteria

Studies that examined the relationship between placenta-derived biomarkers in the context of adverse neurodevelopmental outcomes during the perinatal period in human subjects and written in English were included. Any type of review article, case reports, or editorials were excluded.

As defined by the U.S. Food and Drug Administration and National Institutes of Health (FDA-NIH) Joint Leadership Council, biomarkers are any measured biological end-point which, depending on their source, can be molecular, histologic, radiographic, or physiologic (FDA-NIH Biomarker Working Group, 2016). Biomarkers are used for a myriad of purposes, including diagnostics, disease prediction, and prognostics; thus, we kept our definition of biomarkers open to allow for a wide range of investigations within neuroplacentology.

Maternal conditions (e.g., preeclampsia, diet, stress), delivery complications, and environmental toxicants (e.g., lead, mercury) are exposures or risk factors that may drive placental dysfunction, but are not biomarkers themselves. Studies focused solely on these exposures were excluded in order to reduce confounding factors impacting neurodevelopmental outcomes in the neonate.

Several placental biomarkers associated with maternal conditions (e.g., PIGF for preeclampsia) have been clinically implemented, but diagnostic biomarkers specifically predictive of neurodevelopmental delay remain largely unexplored. Additionally, the goal is to examine signals originating from the placenta, so studies that directly tested for infectious organisms or environmental pollutants were excluded as well.

### 2.2 Data extraction

The final search results were exported into Zotero and duplicates were removed by the application. Titles, abstracts and full texts were screened by two investigators (PP and JK). In an effort to increase consistency among reviewers, both reviewers screened the same records. Sequential evaluation of titles, abstracts, and full texts of all identified publications was conducted, with any disagreements on study selection resolved by consensus between the two reviewers.

## 3 Results

**FIGURE 1:**
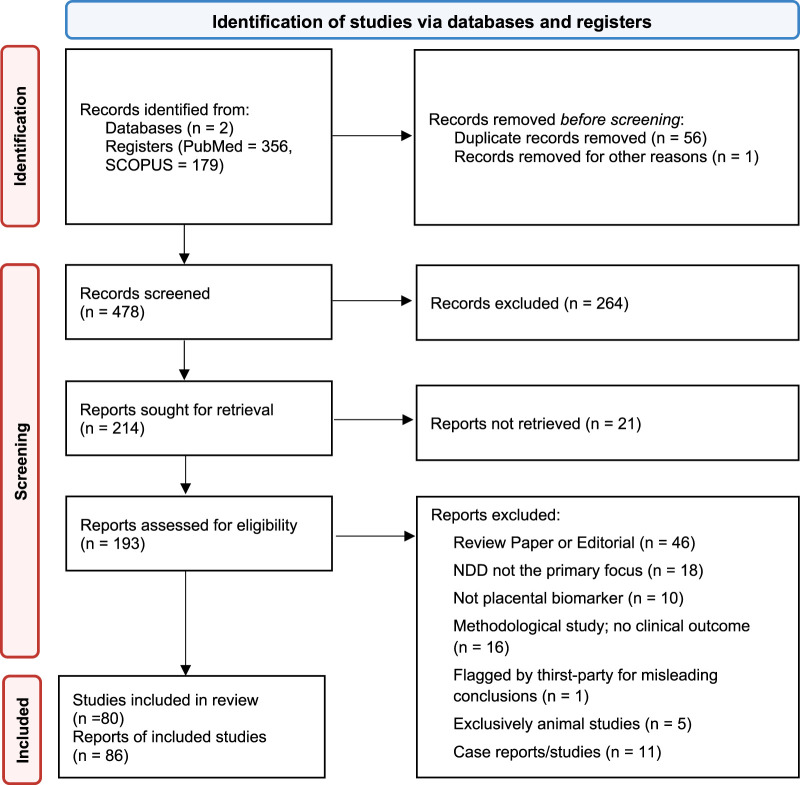
Preferred Reporting Items for Systematic Reviews and Meta-Analyses for Scoping Reviews (PRISMA-ScR) flow diagram.

The initial search criteria outlined above yielded 535 studies, which were reduced to 479 after removing duplicates, 221 after title and abstract screening, and ultimately 106 following review of full texts for study methods. A detailed breakdown is reported in the PRISMA flow diagram Figure 1. We grouped the studies by the type of neurodevelopmental disorder (NDD) they analyzed, organized by date of publication, and summarized the studied biomarker, outcome measures, and key results for each study.

### 3.1 Trisomy 21 - A case study for placental biomarker-based screening

Trisomy 21 (T21) is one of the most commonly screened prenatal conditions, with the American College of Obstetricians and Gynecologists recommending that all pregnant individuals, regardless of age or risk factors, be offered screening and diagnostic testing for T21 (Mj et al., 2022). With over 40 years of collaboration by teams across the world, prenatal testing for T21 can be seen as a model for the progression and development of prenatal screening for NDDs. In addition, almost half of the studies identified in this review are pertaining to T21, with a few also describing the use for other trisomies (T13, T18) (Pertl et al., 1999; Jauniaux et al., 2000; Metzenbauer et al., 2002). As such, we will focus on describing developments within biomarkers for T21 separately from other neurodevelopmental disorders.

Beginning in 1984, initial trials identified several markers with abnormal expression levels in T21 pregnancies that could be captured via urine or maternal serum, including alpha-fetoprotein (AFP), PAPP-A (pregnancy associated plasma protein A), unconjugated estriol 3 (UE3), human chorionic gonadotropin (CG) and free β-hCG (CGB) (Brock et al., 1990; Brizot et al., 1995; David et al., 1996; Newby et al., 1996; Cuckle et al., 1999). Other markers that were investigated can be found in Table 1 (Brock et al., 1990; Newby et al., 2000; Christiansen et al., 1999). Teams also recognized the importance of distinguishing between two prenatal screening periods: first trimester (8–14 weeks) and second trimester (14–18 weeks). Many earlier studies focused on evaluating biomarkers in the second trimester as expression differences between T21 and normal pregnancies were more detectable.

**TABLE 1:** Summary of biomarkers discussed for use in T21 screening (Mj et al., 2022; Pertl et al., 1999; Jauniaux et al., 2000; Metzenbauer et al., 2002; Brock et al., 1990; Brizot et al., 1995; David et al., 1996; Newby et al., 1996; Cuckle et al., 1999; Newby et al., 2000; Christiansen et al., 1999), (Palomaki et al., 2004; Wald et al., 2004; Weinans et al., 2005; Palomaki et al., 2005; Thirunavukarasu et al., 2002; Chim et al., 2008; Du et al., 2011; Zhang et al., 2011; Eckmann-Scholz et al., 2012; Jin et al., 2013; Yin et al., 2014; Lee et al., 2016; Lim et al., 2016; Lim et al., 2017; Lim et al., 2018), (Gordevičius et al., 2020; Lim et al., 2015; Svobodov et al., 2016; Newby et al., 1997; Massin et al., 2001; Maymon et al., 2002; Farina et al., 2003; Prusa et al., 2003; Frendo et al., 2004; Banerjee et al., 2005; Go et al., 2007; Laigaard et al., 2006; Klugman et al., 2008; Papadopoulou et al., 2008; Linskens et al., 2009; Qureshi et al., 1997; Sun et al., 2011; Chen et al., 2012; Munnangi et al., 2014; Pinilla et al., 2015; Wong et al., 2018).

Paper	Biomarker
Brock et al. (1990)	AFP, UE3 , CG, SP1, PLAP
Brizot et al. (1995)	CGA, CGB
David et al. (1996)	UE3
Newby et al. (1996)	CG, CGB, SP1, PLAP, AFP, GGT
Newby et al. (1997)	CG, CGB, SP1, PLAP, AFP, GGT, PAPP-A
Christiansen et al. (1999)	proMBP
Cuckle, H. S., Canick, J. A., and Kellner, L. H. (1999)	CGB
Pertl et al. (1999)	Trisomy 21: *D21S11*, *D21S1411*, *D21S1412*, and *D21S1414* Trisomy 18: *D18S535*, STRs from the myelin basic protein gene (MBP), and *D18S386* Trisomy 13: *D13S631*, *D13S634*, and *D13S258*
Jauniaux et al. (2000)	CGB
Newby et al. (2000)	uE3, DHEAS, STS
Massin et al. (2001)	CGA, CGB
Maymon et al. (2002)	p43-PLF
Metzenbauer et al. (2002)	Placental volume
Thirunavukarasu et al. (2002)	Inhibin A (INHA and INHBA)
Farina et al. (2003)	cffDNA
Prusa et al. (2003)	hTERT, LIFR, BMPR2
Sutton and Cole (2004)	ITA
Frendo et al. (2004)	CG
Palomaki et al. (2004)	ITA
Wald et al. (2004)	PAPP-A, CGB, CG, UE3, AFP, ITA, CG, CGB
Banerjee et al. (2005)	CGB and *LHCGR*
Palomaki et al. (2005)	ITA
Weinans et al. (2005)	ITA
Go et al. (2007)	*C21orf105*
Laigaard et al. (2006)	ADAM12
Chim et al. (2008)	Differential methylated CpG sites on Chromosome 21, *U-PDE9A*, *U-CGI137*
Klugman et al. (2008)	TRAIL, KRT8
Papadopoulou et al. (2008)	hGPH
Linskens et al. (2009)	CGB, PAPP-A
Qureshi et al. (1997)	PCNA
Du et al. (2011)	*AIRE*, *POTE*, *DSCR4*
Sun et al. (2011)	SOD1, ERp29, HSP27, PRDX6
Zhang et al. (2011)	*AIRE*, *RASSF1A*
Chen et al. (2012)	Previously reported: AAT, CRYAA, APOA1, CTSD, HSPB1, MDH, MBP, PRDX2, PRDX6, SAP, TCP1, TTR, VIM, VDAC2 New: ASB17, ANXA2, ANXA5, ATXN3, CALR, CSH1, PPIB, XRCC2, EVPL, LPLA2, BLVRB, GRK4, LGALS1, GBP2, KRT8/18, KRT222, KLR, GLO1, ACP1, MPP1, MSP1, MRPP2, PFN1, P4HB, RCN1, NEK7, SRI, SPRED2, VIPR1
Eckmann-Scholz et al. (2012)	27,578 differentially methylated CpG sites from more than 14,000 genes
Jin et al. (2013)	*TET1*, *TET2*, *REST*
Lim et al. (2015)	34 miRNAs of placental origin
Munnangi et al. (2014)	PAPP-A2
Yin et al. (2014)	*CGI149*, *CGI045*, *HLCS-1*, *HLCS-2*
Pinilla et al. (2015)	CBS
Lee et al. (2016)	*FSMR-E*, *FSMR-U1*, *FSMR-U2*
Lim et al. (2016)	DNA methylation patterns of 207 genes on Chromosome 21
Svobodová et al. (2016)	754 miRNAs of placental origin
Lim et al. (2017)	110 candidate genes in whole genome
Lim et al. (2018)	110 candidate genes in whole genome
Wong et al. (2018)	APP, ETS2, SOD1, HMGN1
Lim et al. (2019)	DNA methylation of CpG sites distributed across the whole genome
Leon-Martinez et al. (2020)	CASP2

Entering the new millennium, invasive trophoblast antigen (ITA) and inhibin A were introduced as a new potential biomarker for T21, reflecting the ongoing efforts at the time in improving the sensitivity and specificity of prenatal T21 screening (Sutton and Cole, 2004; Palomaki et al., 2004; Wald et al., 2004; Weinans et al., 2005; Palomaki et al., 2005; Thirunavukarasu et al., 2002). At the same time, there was an increasing focus on the optimization of screening protocols, with Wald et al. as seen in Table 1 comparing false positive rates for several combinations of biomarkers that have since helped to guide current screening procedures as will be described below (Wald et al., 2004).

Since then, investigations have shifted from protein-based methods towards epigenetic-based methods to improve noninvasive T21 prenatal screening (Chim et al., 2008; Du et al., 2011; Zhang et al., 2011; Eckmann-Scholz et al., 2012; Jin et al., 2013; Yin et al., 2014; Lee et al., 2016; Lim et al., 2016; Lim et al., 2017; Lim et al., 2018; Lim et al., 2019; Gordevičius et al., 2020). Differentially methylated DNA is used in the identification and isolation of cffDNA from maternal serum samples, with teams identifying several CpG sites which were specifically hyper- or hypomethylated in fetal samples, allowing for noninvasive prenatal screening using cffDNA. The upregulation of certain microRNAs (miRNAs) was also highlighted by several teams as potential screening tools for T21 (Lim et al., 2015; Svobodov et al., 2016).

**FIGURE 2:**
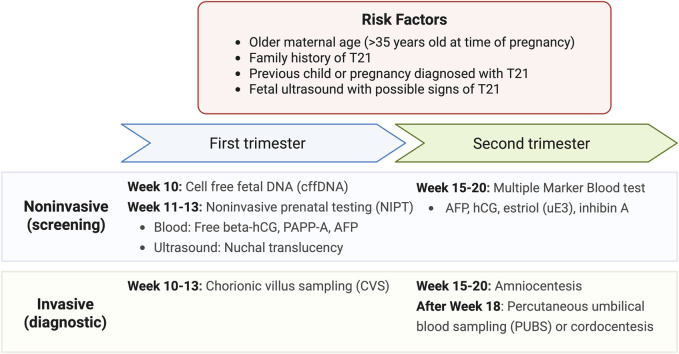
Current tests available for Down Syndrome screening and diagnosis. Results from noninvasive screening from first and second trimesters can also be integrated into the combined testing method which provides a more robust estimate of the risk of prenatal T21 (MedlinePlus Medical Test, 2025).

At this time, prenatal T21 testing includes noninvasive screening and invasive diagnostic tests as illustrated in Figure 2 (MedlinePlus Medical Test, 2025). These are typically performed for expectant mothers with risk factors such as older maternal age (>35 years of age at time of pregnancy), family history of T21, having a previous child or pregnancy diagnosed with T21, and a fetal ultrasound with possible signs of T21. Looking to the future, it continues to be important to improve the cost-effectiveness of testing while reducing the rates of false positives. This demonstrates the process of developing minimally invasive and high specificity diagnostic tools to improve neurological outcomes in fetuses and neonates–a process that will continue to evolve for other neurodevelopmental outcomes described below.

### 3.2 Current investigations

**FIGURE 3:**
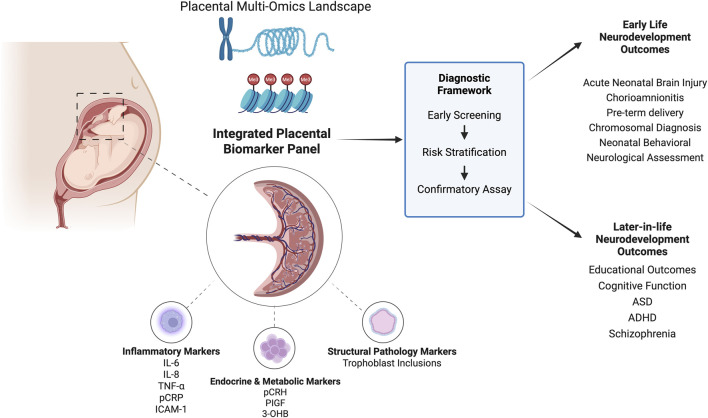
Landscape of placental biomarkers for early screening of neurodevelopmental outcomes. Review shows potential of an integrated placental biomarker panel to develop a diagnostic framework similar to Trisomy 21.

As discussed, the placenta also modulates numerous interactions across the maternal-fetal axis. Current studies on placental biomarkers highlight their diverse roles in inflammation, hypoxic signaling, timing of delivery, hormone and metabolic regulation, and various omics-related pathways as listed in Table 2. Beyond established use in T21 and select genetic disorders (e.g. T13, T18), placental biomarkers for other neurodevelopmental outcomes remain exploratory and have yet to enter routine clinical practice. Figure 3 highlights the promise of an integrated biomarker approach in this expanding area of research.

**TABLE 2:** Summary of exploratory placental biomarkers with current limitations and future directions.

Category	Biomarkers	Limitations/Future directions
Inflammatory	IL-6, IL-8, G-CSF, TNF-α, ICAM-1 CRP GFAP, UCH-L1, LC3 Adrenomedullin mRNA	Low specificity due to overlap with multiple inflammatory states, often studied in small cohorts. External validation is needed to establish predictive value and support integration into routine perinatal testing
Metabolic/Growth	pCRH Placental Growth Factor (PlGF) Placental weight 3-hydroxybutyrate (3-OHB) APOE	Associations are time specific and based on small or demographically narrow cohorts. Larger, more diverse studies are required with emphasis on combining metabolic signals into biomarker panels
Omics/Epigenetic	pDMRs Gene expression Fragile X (FMR1 inactivation timing) Integrated multi-omics (mRNA + miRNA + methylation)	High heterogeneity and lack of standardization limit reproducibility, with most findings still in the discovery phase. Multi-omics approaches show promise but require large multicenter validation before clinical application

#### 3.2.1 Inflammatory markers

Over the past few decades, studies have made significant strides from identifying inflammatory markers during pregnancy to linking distinct inflammatory pathways with specific NDDs. In 1992, Saito et al. identified elevations in cytokines (IL-6 and IL-8) in cytotrophoblasts and decidual stromal cells in cases of chorioamnionitis and preterm births, implicating intrauterine infection in neonatal outcome (Saito et al., 1993). These early observations prompted researchers to ask whether inflammatory markers implicated in preterm birth might also be involved in fetal brain injury, thereby linking placental inflammation to the pathogenesis of neonatal encephalopathy (NE). NE typically manifests with difficulty initiating breathing, low muscle tone, decreased consciousness, and seizures. It often occurs as a complication of perinatal hypoxia and/or pro-inflammatory cytokines, which impair gas and nutrient exchange and cause oxidative stress. It is associated with increased risk of death and long-term neurodevelopmental impairment, thus is a significant area of perinatal research (Shankaran et al., 1991; Kurinczuk et al., 2010).

Early explorations into genetic susceptibility identified HLA allele subtypes, namely, HLA-B, -DR, and -DQ, as a potential risk factor for NE (Cowan et al., 1994). Expansion of the placental landscape has identified glial fibrillary acidic protein (GFAP), an intermediate filament protein in astrocytes; ubiquitin carboxy-terminal hydrolase L1 (UCH-L1), a neuroendocrine specific enzyme; and increased LC3 expression in syncytiotrophoblasts, a marker of autophagy, as promising biomarkers for detecting the incidence and severity of NE. (Mir and Chalak, 2014; Douglas-Escobar et al., 2010; Avagliano et al., 2013).

Several studies have examined the timing and maternal context of inflammatory responses, including distinct oxidative stress signatures in fetal brain versus placental samples and adrenomedullin mRNA levels in the placenta to pinpoint the timing of hypoxic-ischemic encephalopathy associated death (Baldari et al., 2023). The expression of peptide adrenomedullin by fetal trophoblasts has been studied as a potential biomarker and therapeutic agent for preeclampsia primarily in animal models due to its role in the maternal-fetal perfusion response (Zhang et al., 2023; Li et al., 2013). Adrenomedullin mRNA in humans provides a window into timing that expands upon well-established and routine markers like pH and lactate levels to differentiate between acute and chronic intrauterine hypoxia; however, its potential as a prognostic factor needs to be explored (Trollmann et al., 2002; Gea et al., 2007).

**FIGURE 4:**
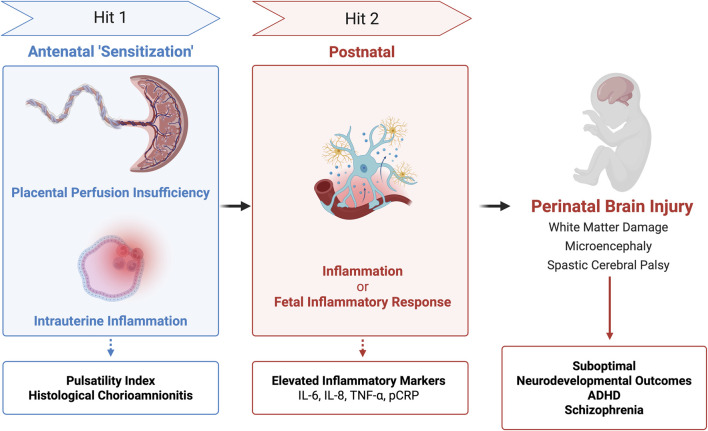
Two Hit Hypothesis of Perinatal Brain Injury. Integration of placental testing (pulsatility index, histology, and inflammatory markers) based on timing of injury (‘hits’) may have associations with brain injury and long-term neurodevelopmental outcomes.

Timing of inflammatory responses has brought about the two-hit hypothesis which, as shown in Figure 4, proposes that sequential antenatal and postnatal inflammatory insults (‘hits’) contribute to neurodevelopmental injury. Mapping risk profiles across the placenta began with the umbilical cord using new screening tools and imaging techniques. Studies reported that isolated placental perfusion insufficiency, detected antenatally by elevated pulsatility indices in the umbilical artery, ductus venosus, and inferior vena cava, predicted suboptimal neurodevelopmental outcomes at 1 year even when histological inflammation was absent (Kaukola et al., 2005).

Subsequent work broadened the model to intrauterine inflammation. Some cases of histological chorioamnionitis (HCA) progress to fetal inflammatory response (FIR) and at its extreme to fetal inflammatory response syndrome (FIRS). Conversely, non-infectious stressors like hypoxia-ischemia can trigger FIR/FIRS in the absence of HCA. These infectious or sterile pathways describe two related intrauterine ‘hits’ in preterm infants. Regardless of the initiating pathway, elevated cytokine levels (IL-6, IL-8, G-CSF) in the umbilical cord correlated with increased severity of brain injury (Lu et al., 2016). Some researchers speculated that the first ‘hit’ may be sensitizing the fetal brain to subsequent perinatal or postnatal stressors.

Yanni et al. explicitly applied the two-hit framework and demonstrated that antenatal inflammation (perfusion insufficiency or HCA) followed by postnatal inflammation (elevated inflammatory proteins) significantly increased the risk of adverse neurodevelopmental outcomes. Specifically, elevated placental C-reactive protein (pCRP) and ICAM-1 were associated with white matter damage; TNF-α and IL-8 with spastic cerebral palsy; and IL-6, TNF-α, or ICAM-1 with microcephaly. These findings suggest cytokines are not merely messengers of inflammation, but may drive or even directly mediate damage to neural tissue, making them potential candidates for perinatal screening or diagnostic biomarkers (Yanni et al., 2017). However, further research is needed to improve their specificity and diagnostic accuracy. Other investigations have linked pregnancy-related anxiety to attention-deficit/hyperactivity disorder (ADHD) symptoms at 36 months of age with sex-specific associations, particularly in boys, between pCRP mRNA expression, TNF-α and ADHD symptoms (Shao et al., 2020). Larger cohorts have expanded into neuropsychiatry, namely, schizophrenia, in which each 1 mg/L increase in maternal CRP was associated with a 28% higher risk of schizophrenia (Canetta et al., 2014). Collectively, these findings support that the placenta archives fetal exposure to infection, hypoxia, and oxidative stress. Integrating cytokines, acute phase reactants, placenta-derived peptides and autophagy markers into a biomarker panel could enable early risk stratification of infants at risk and shift perinatal clinical practice toward precision medicine.

#### 3.2.2 Metabolic markers

Placental biomarker research has gone beyond acute injury to investigating placenta as a predictive organ for long-term neurodevelopmental outcomes. Endocrine and growth-related signals are emerging as especially informative. Elevations in placental corticotropin-releasing hormone (pCRH) have been associated with poor motor outcomes and ventriculomegaly with a study finding that the timing of pCRH exposure at 19 weeks and 31 weeks altered cortical development and spatial attention, respectively (Sandman et al., 2018; Leviton et al., 2016).

Studies found that fetal growth restriction (FGR) and placental growth factor (PlGF) in the second trimester were predictive of lower educational outcomes at 5–7 years and neurodivergent behaviors, respectively (Leon-Martinez et al., 2020; Tsompanidis et al., 2023). The study also found that distinct PlGF patterns were associated with male sex, potentially explaining the higher prevalence of neurodivergent behaviors observed in males.

Decreased placental weight has been connected to increased risk of FGR for several years. More recently, the opposite end has been explored with one study reporting a possible link between increased placental weight and risk of Autism Spectrum Disorder (ASD). However, placental weight is a nonspecific biomarker and the study relied on a small subset of single nucleotide polymorphisms; thus, placental weight may be a potential proxy for intrauterine conditions rather than a causal mechanism (Liu et al., 2021; Liu, 2024).

In parallel, emerging work expands the scope of placental biomarkers to metabolic signals, namely, 3-hydroxybutyrate (3-OHB). 3-OHB, a maternally produced ketone body, crosses the placenta and is a signal of metabolic stress. The included study reported that elevated umbilical cord 3-OHB levels were linked to atypical development on the Mullen Scales of Early Learning, but showed no association with ASD (Parenti et al., 2024). Further exploration into metabolomics as a diagnostic tool in addition to growth factors and placental weight may be a powerful combination for risk stratification of long term neurodevelopmental outcomes.

#### 3.2.3 Vast omics of the placenta

This expanding view reflects a broader shift toward the omics of the placenta to create a more integrated picture of fetal programming as shown in Figure 3.

Analysis of ‘omics’ emphasizes the placenta as a ‘record-keeper’ of the fetal environment with research progressing from broad measures like differentially methylated regions in the placenta (pDMRs) and isolated gene expression to an integrated, multi-omics approach.

Earlier studies focused on imprinted gene expression with outcomes showing distinct placental expression profiles. In Fragile X syndrome, the Fragile X Messenger Ribonucleoprotein 1 (FMR1) gene in chorionic villi samples showed absence of FMR1 in cytotrophoblasts and connected gestational age to the timing of inactivation of the FMR1 allele with DNA methylation found at specific CpG islands (Willemsen et al., 2002; Luo et al., 1993). These studies demonstrated a new method for prenatal diagnosis of Fragile X syndrome by leveraging placental expression and epigenetic timing; however, it has yet to be clinically implemented.

Building on this foundation, research has increasingly focused on the prenatal origins of ASD, linking it to maternal immune activation, gestational diabetes, and metabolic conditions (Liu, 2024; Li et al., 2016; Meltzer and Van de Water, 2017; Gardner et al., 2015; Xiang et al., 2015). Structural abnormalities in the placenta, such as trophoblast inclusions, have been associated with altered neurodevelopment, though causative relationships in placental structure continue to be explored (Anderson et al., 2007). Epigenetic studies further support placental involvement, with both hyper- and hypomethylated CpG regions linked to ASD, attention deficits, and cognitive delays. Hypermethylated CpG regions associated with reduced expression of GPR135, ITGBL1, FHIT, IRS2, DLL1, and LRRFIP1 suggest disruptions in synapse quantity, neuritogenesis, abnormal morphology, immune regulation, and GABAergic neuronal signaling, while hypomethylated regions affecting CYP2E1 and 22q13.33 imply dysregulation in oxidative stress and synaptic development/function (Bahado-Singh et al., 2021; Paquette et al., 2016; Tilley et al., 2018; Zhu et al., 2019; Zhu et al., 2022; Schroeder et al., 2016; Freedman et al., 2023).

As the understanding of the perinatal environment develops, research has moved beyond broad methylation profiles toward gene-specific signals. Several placental proteins and epigenetic marks have emerged as early indicators of later neurodevelopment. One team found Apolipoprotein E (APOE) to be upregulated in the placenta of preterm infants who experienced developmental delays at 6 months (Zhu et al., 2023). APOE, a key mediator of lipid transport and synaptic plasticity, complements the metabolomic finding of 3-OHB by underscoring the notion that placental lipid handling shapes early neurodevelopment and offers promises as a screening biomarker (Lane-Donovan and Herz, 2017).

Single-cohort reports show that methylation of neuronal growth regulator 1 (NEGR1) and serotonin 2A receptor (HTR2A) correlated with adverse developmental outcomes, but many of these findings await confirmation in subsequent studies (Breton et al., 2020; Paquette et al., 2013). Integrating multiple omic layers can markedly enhance biomarker performance. Santos et al. found integration of methylation, mRNA, miRNA data improved accuracy of predicting ASD compared to single platform model (Santos et al., 2020).

## 4 Discussion

Neuroplacentology is a rapidly growing field and stands to provide incredible improvements in risk stratification for pregnant women and neonates. For instance, a team from the University of Texas Southwestern’s Parkland Hospital has implemented a targeted hospital-based protocol for triaging placentas from high-risk deliveries for histological evaluations in order to inform and facilitate timely treatment intervention strategies (Mir et al., 2021). This demonstrates the promising future of neuroplacentology and its possible incorporation into hospital-based practices in order to better address adverse neurodevelopmental outcomes that are amenable to early intervention, such as neonatal encephalopathy and chorioamnionitis.

As screening methods within neuroplacentology continue to improve, the psychosocial welfare of mothers must continue to be a main consideration. In 2017, the Netherlands launched a nationwide study known as the TRIDENT-2 study which offered NIPT to all pregnant women (van der Meij et al., 2019). The study focused on screening for T13, T18, T21, with additional findings outside of these three conditions being provided only upon request of the mother. Six years later, results on the psychological impacts of the testing’s “additional findings” were discussed by Bakkeren et al. (2024). Over 92% of women stated that receiving the additional finding was an unexpected shock and 85% reported that it caused significant amounts of worry. The study also looked at measures of anxiety and distress, highlighting that women who gave birth to an affected child were most impacted.

Currently, the American College of Obstetricians and Gynecologists (ACOG) recommends that all pregnant women be offered the option of screening and diagnostic testing for Down syndrome. As NIPT becomes more accessible to mothers across the globe, healthcare providers must remain vigilant in supporting women to understand the implications of prenatal testing (Screening for Fetal Chromosomal Abnormalities, 2020). This includes the potential need for and risks of follow-up invasive diagnostic tests. Counseling plays an important role in managing expectations and concerns regarding testing results, and should be strongly recommended to mentally prepare women for the possible outcomes from these screenings.

Additionally, it would be remiss not to mention that the literature examined in this review comes predominantly from high-income countries. As such, prediction models built upon data generated by these studies may not be validated in low- to middle-income countries. Antwi et al. share a possible method of addressing this challenge, such as updating the models to align with clinical characteristics observed within that population (Antwi et al., 2020). It is also important to recognize that serum-based biomarker screenings may not be as readily available in these countries as well, which impacts the generalizability of results and outcomes regarding the clinical implementation of placental biomarker-based screenings.
