# Supplementary material for: Placental biomarkers for the prediction of neurodevelopmental disorders
Source: Front Cell Dev Biol. 2025 Oct 7;13:1663960. doi: 10.3389/fcell.2025.1663960 (PMC12537792; doi:10.3389/fcell.2025.1663960)
Supplement: Supplementary file 1 [file Table1.docx]

**PUBMED SEARCH CRITERIA**

(("Biomarkers"[Mesh] OR "Genomics"[Mesh] OR "Metabolomics"[Mesh] OR "Epigenesis, Genetic"[Mesh] OR "MicroRNAs"[Mesh] OR "Cytokines"[Mesh]

OR "biological correlates"[Title/Abstract] OR biomarker*[Title/Abstract] OR "biological marker"[Title/Abstract] OR "biological markers"[Title/Abstract]

OR "inflammatory marker"[Title/Abstract] OR "inflammatory markers"[Title/Abstract] OR "genetic marker"[Title/Abstract] OR "genetic markers"[Title/Abstract]

OR Genom*[Title/Abstract] OR Metabolom*[Title/Abstract] OR Epigen*[Title/Abstract] OR MicroRNA*[Title/Abstract] OR Cytokine*[Title/Abstract])

AND

("Placenta"[Mesh] OR "Placental Diseases"[Mesh] OR "Maternal-Fetal Exchange"[Mesh] OR "Placental Circulation"[Mesh]

OR "placental biomarkers"[Title/Abstract] OR "maternal vascular malperfusion"[Title/Abstract] OR "fetal vascular malperfusion"[Title/Abstract]

OR "acute chorioamnionitis"[Title/Abstract] OR "chronic villitis"[Title/Abstract] OR "villitis of unknown etiology"[Title/Abstract])

AND

("Neurodevelopmental Disorders"[Mesh] OR "Brain Diseases"[Mesh] OR "Child Development"[Mesh] OR "Neurologic Manifestations"[Mesh]

OR "neurodevelopmental outcomes"[Title/Abstract] OR "neurodevelopmental diseases"[Title/Abstract] OR "neuroplacentology"[Title/Abstract]

OR "neuronal development"[Title/Abstract] OR "neonatal encephalopathy"[Title/Abstract] OR "neonatal neurologic morbidity"[Title/Abstract])

NOT

("Animal Models"[MeSH] OR "Non-Human"[MeSH] OR "Animals"[Title/Abstract] OR "Animal Studies"[Title/Abstract])

NOT

("Maternal Nutritional Physiological Phenomena"[Mesh] OR "Preeclampsia"[Mesh] OR "Hypertension, Pregnancy-Induced"[Mesh]

OR "Heart Defects, Congenital"[Mesh] OR "Cesarean Section"[Mesh] OR "Placental Abruption"[Mesh] OR "Postpartum Hemorrhage"[Mesh]

OR "maternal diet"[Title/Abstract] OR "preeclampsia"[Title/Abstract] OR "hypertension"[Title/Abstract] OR "delivery complications"[Title/Abstract]

OR "congenital heart defects"[Title/Abstract] OR "C-section"[Title/Abstract] OR "placental abruption"[Title/Abstract] OR "hemorrhage"[Title/Abstract])

**SCOPUS SEARCH CRITERIA:**

(TITLE-ABS-KEY("biological correlates" OR biomarker* OR "biological marker" OR "biological markers"

OR "inflammatory marker" OR "inflammatory markers" OR "genetic marker" OR "genetic markers"

OR Genom* OR Metabolom* OR Epigen* OR MicroRNA* OR Cytokine*)

AND

TITLE-ABS-KEY("placental biomarkers" OR "maternal vascular malperfusion" OR "fetal vascular malperfusion"

OR "acute chorioamnionitis" OR "chronic villitis" OR "villitis of unknown etiology" OR Placenta OR "Placental Diseases"

OR "Maternal-Fetal Exchange" OR "Placental Circulation")

AND

TITLE-ABS-KEY("neurodevelopmental outcomes" OR "neurodevelopmental diseases" OR "neuroplacentology"

OR "neuronal development" OR "neonatal encephalopathy" OR "neonatal neurologic morbidity" OR "Neurodevelopmental Disorders"

OR "Brain Diseases" OR "Child Development" OR "Neurologic Manifestations"))

AND

NOT TITLE-ABS-KEY("Animal Models" OR "Non-Human" OR "Animals" OR "Animal Studies")

AND

NOT TITLE-ABS-KEY("maternal diet" OR "preeclampsia" OR "hypertension" OR "delivery complications"

OR "congenital heart defects" OR "C-section" OR "placental abruption" OR "hemorrhage" OR

"Maternal Nutritional Physiological Phenomena" OR "Preeclampsia" OR "Hypertension, Pregnancy-Induced"

OR "Heart Defects, Congenital" OR "Cesarean Section" OR "Placental Abruption" OR "Postpartum Hemorrhage")
