# Supplementary material for: Placental biomarkers for the prediction of neurodevelopmental disorders
Source: Front Cell Dev Biol. 2025 Oct 7;13:1663960. doi: 10.3389/fcell.2025.1663960 (PMC12537792; doi:10.3389/fcell.2025.1663960)
Supplement: Supplementary file 2 [file DataSheet1.pdf]

Authors	Biomarker	Outcome Measure	N (Samples/Cohort/Sample Source)
Saito et al. (1993)	IL-6, IL-8 & G-CSF in the Amniotic Fluid	CAM and Preterm Delivery	*Amniotic Fluid of Preterm Delivery*<br>Group 1: CAM (-) N = 20<br>Group 2: CAM (+) N = 31<br><br>*Amniotic Fluid of Term Delivery*<br>Group 3 Pain (-)/Endotoxin (-) N = 20<br>Group 4 Endotoxin (-) N = 20<br>Group 5 Pain (+) Endotoxin (+) N =12
Firestein et al. (2017)	Trophoblast Inclusions (TI)	GA at Birth	N = 108 infants with at least four placental histology sldies containing chorionic villi (26-34 weeks gestational age)
Olga et al. (2023)	Antenatal fetal growth restriction (FGR; fetal weight < 10th percention @ 36 wks)	Education outcomes @ ages 5-7 yrs	Group 1: FGR N = 250<br>Group 2: Appropriate for gestational age (AGA) with placental dysfunction N =949<br>Group 3: healthy small-for-gestational age (SGA) N = 126<br>Group 4: healthy AGA N = 1429
Zhu et al. (2023)	MLPT & PGE	ASQ-C @ 6, 18, 48 months	MLPT group N = 129<br>Term group N = 3136
Cheng et al. (2025)	PGE	ASQ-C @ 6, 18, 48 months	Small-for-gestational age [SGA] N = 316<br>Appropriate for gestational age [AGA] N = 2406

Sandman et al. (2017)	pCRH	Cortical Thickness on MRI	N = 97; 49 girls
Shao et al. (2020)	Pregnancy-Related Anxiety Questionnaire (PRAQ) and Cytokines [IL01B, IL-6, IL-8, TNF-a, CRP]	ADHD symptoms using Conners' Hyperactivity Index	N = 2926 [1173 no preg-related anxiety; 510 at least one trimester; 685 at least 2 trimester]
Schroeder et al. (2016)	pDMR	ADOS-G and ASD DSM-5 diagnosis	N = 24 ASD<br>N = 23 typically developing TD
Zhu et al. (2019)	pDMR	ASD DSM-5 diagnosis	N = 41; (20 ASD & 21 TD)
Santos et al. (2020)	pDMR and PGE	ASD SCQ and ADI-R and DAS-II for IQ assessment	N = 379 (N = 35 ASD cases, 9.3% of the sample) placentas from children diagnosed with ASD (N = 24) compared to placentas from typically developing (N = 23) children
Bahado-Singh et al. (2021)	pDMR	ASD DSM-5 diagnosis	14 term autism cases (7 males, 7 females) and 10 term ethnicity-matched normal controls (5 males, 5 females)
Zhu et al. (2022)	pDMR and PGE	ASD diagnosis	MARBLE (ASD n = 21, Non-TD n = 13, TD n = 31) TD (typical neurdevelopement)<br>EARLI (ASD n = 16, TD n = 31)

Freedman et al. (2023)	pDMR and PGE	ASD diagnosis with SCQ and ADI-R @ age 10	N - 368 (ASD n = 28; non-ASD n = 340)
Tsompanidis et al. (2023)	PGE	ASD	N - 39 (N = 17 Female, N = 22 Males)
Tsompanidis et al. (2023)	Placental growth factor (PIGF), SFLT-1 in maternal plasma in 1st and 2nd trimester	ASD DSM-5 diagnosis and SRS	N = 1290 Males with no autistic traits<br>N = 1356 Males with placental markers and autistic traits<br>N = 1314 Females with missing autistic traits<br>N = 1254 females with placental markers and autistic traits
Liu, Zhao (2024)	Placental Weight and SNPs of PW	ASD	Early Growth Genetics (EGG) Consortium and ASD GWAS
Parenti et al. (2024)	Cord Serum and Placental 3-hydroxybutyrate (3-OBH)	Autism Diagnositc Observation and Mullen Scales of Early Learning	ASD n = 45, Non-TD n = 19, TD n = 87) TD (typical neurdevelopement)
Leviton et al. (2016)	pCRH mRNA	Brain Ultrasound and Bayley Scales of Infant Development II scores @ 24 months	N = 1243 (Born before 28th week of gestation)
Yanni et al. (2017)	Antenatal Inflammation and<br>Postnatal inflammation	Brain Ultrasound and Bayley Scales of Infant Development II scores @ 24 months	n = 763

Lu et al. (2016)	CAM and cytokines in umbilical cord	Brain Injury on Imaging (Ultrasound and/or MRI)	N = 103 infants with PPROM (preterm premature rupture of membranes)<br>HCA (-)FIRS(-) n = 48<br>HCA (+)FIRS(-) n = 34<br>HCA (+) FIRS (+) n = 21
Cowan et al. (1994)	HLA antigens in venous blood samples	NE	Modeterate to Severe encephalopathy n = 13<br>Seizure with no encephalopathy n = 7<br>Control infants n = 20
Avagliano et al. (2013)	Placental Immunostaining	NE	Neonatal Encephalopathy n = 6<br>Controls n = 34
Mir et al. (2019)	GFAP and UCH-L1 in UmA and UmV	NE	Group 1: Uncomplicated c-delivery w/o labor n = 15<br>Group 2: Uncomplicated vaginal delivery w/ labor n = 15<br>Group 3: Perinatal hypoxia-asphyxia+NE n = 8
Willemsen et al. (2002)	FMRP expression	timing of gestation?	17 FM male fetuses; Two cases of FM female fetuses
Trollmann et al. (2002)	Adrenomedullin (ADM) mRNA during first 12h of life	Severity of birth asphyxia	HIE 0/1 n = 12<br>HIE 2/3 n = 5<br>Controls n =10
Kaukola et al. (2005)	Inflammatory markers and VEGF in umbiliacal cord	Griffiths Scales at 1 year of corrected age	Group 1 (Suboptimal outcome) with placenta perfusion insufficiency n = 7<br>Group 2 (normal outcome) with placenta perfusion insufficiency n = 10<br>HCA placenta n = 25<br>Normal placenta n = 10

Gea, Yvi, Araujo, Orlei, and Silva, Luiz Vicente R. (2007)	Lactate and nucleated red blood cells (NRBC)	Hypoxia-Ischemia in Premature infants	n = 25 umbilical cords premature babies
Baldari et al. (2023)	Oxidative stress markers (NOX2, 8-OHdG, NT, iNOS, IL-6) in brain and placenta	HIE-related death timing	Fetal intrauterine death n = 9<br>Intrapartum death n = 8<br>Post-partum death n = 6<br>Control sudden neonatal death n = 6
Green et al. (2015)	PGE	NNNS	n = 615
Marsit et al. (2012)	PGE	NNNS	n = 106 placentas
Paquette et al. (2013)	Placental methylation of HTR2A	NNNS	n = 444
Paquette et al. (2014)	Placental methylation and PGE of FKBP5	NNNS	n = 61
Torres-Espínola et al. (2015)	Pro12Ala polymorphism on exon 3 of PPARG gene (rs1801282 C/G)<br><br>Placenta Sample with Fatty acid analysis	Bayley Scale of Infant and Toddler Developement III test given @ 6 and 18 months of age [cognitive, language, motor and social development]	N = 138 [CC genotype N = 118 & CG genotype N = 20]
Paquette et al. (2016)	pDMR	NNNS	n = 335
Tilley et al. (2018)	pDMR	Cognitive function at ten years of age DAS-II	n = 84 (n = 59 spontaneous EPTB, n = 25 indicated EPTB)
Breton et al. (2020)	Placental methylation pf NEGR1	SDQ at 3yo and BMI	n = 276 mother-child dyads

Ursini et al. (2021)	Placental GRS	neonatal intracranial volume (ICV) and Mullen early learning composite standardized score (20) at 1 (MCS1) and 2 y of age (MCS2).	n = 242 (n = 147 singletons, n = 95 multiple pregnancies)
Canetta et al. (2014)	Maternal CRP	Diagnosis of schizophrenia (ICD-10 F20) or schizoaffective disorder (ICD-10 F25)	N = 777 (630 cases of schizophrenia, 147 cases of schizoaffective disorder) with matching of 1:1 to controls
Brock et al. (1990)	AFP, uE3 (unconjugated estriol 3), hCG, SP1 (pregnancy-specific beta 1 glycoprotein) and PALP (placental ALKP)	Down Syndrome diagnosis	N = 21 (Down syndrome) with each case matched to three healthy controls of similar gestational age (19 first-trimester and two at 14 weeks) and window of time of sample storage
Brizot et al. (1995)	Alpha and beta subunits of hCG	Fetal Trisomy 21	N = 9 (trisomy 21) and N = 30 (controls), 9 control samples were also matched to trisomy 21 group based on gestational age at time of pregnancy termination
David et al. (1996)	uE3	Down Syndrome diagnosis	Group 1: N = 18764 normal singleton pregnancies only for hCG and AFP, N = 9311 normal singleton pregnancies also screened for additional uE3<br><br>Group 2: 47 women with Down syndrome pregnancies
Newby et al. (1996)	Intact hCG, free beta-hCG, SP1, PALP, AFP, GGT in placental tissue	Down Syndrome diagnosis	N = 67 (Down Syndrome) and N = 75 (Unaffected)
Newby et al. (1997)	Intact hCG, free beta-hCG, SP1, PALP, AFP, GGT, PAPP-A	Down Syndrome diagnosis	N = 67 (Down Syndrome) and N = 75 (Unaffected)
Christiansen et al. (1999)	proMBP	Trisomy 21 karyotyping	First trimester samples (maternal serum via syphilis screening program at Statens Serum Institut, Copenhagen): N = 25 (DS) and N = 156 (normal)<br><br>Second trimester samples (maternal serum via DS and fetal malformation screening program at Statens Serum Institut, Copenhagen): N = 105 (DS) and N = 151 (normal)
Cuckle, H. S., Canick, J. A., and Kellner, L. H. (1999)	Urinary beta-hCG	Down Syndrome diagnosis	N = 6730 pregnancies: 39 with Down syndrome, 12 with Edwards’ syndrome, 42 with other aneuploidies, 52 unaffected twins and 6585 singleton unaffected pregnancies

**Pertl et al. (1999)**	Trisomy 21: D21S11, D21S1411, D21S1412, and D21S1414<br>Trisomy 18: D18S535, STRs from the myelin basic protein gene (MBP), and D18S386<br>Trisomy 13: D13S631, D13S634, and D13S258	Trisomy 13, 18, 21	CVS samples N = 222 (Normal), N = 1 (Trisomy 13), N = 5 (Trisomy 18), N = 15 (Trisomy 21), N = 2 (Mosaic 21) and N = 1 (Mosaic 18)
**Jauniaux et al. (2000)**	β-hCG	Trisomy 21 or Trisomy 18 via karyotyping	Placental tissue samples N = 42 (Normal), N = 8 (T21), N = 6 (T18)
Newby et al. (2000)	uE3, DHEAS, and STS	Down Syndrome diagnosis	N = 92 (Down Syndrome) and N = 76 (Unaffected), includes the existing cohort mentioned in 1996 and 1997
Massin et al. (2001)	Alpha and beta subunits of hCG	Trisomy 21 affected by determination of DNA polymorphism markers or karyotyping	Group 1: First trimester placentae from legal abortion<br>Group 2: Second-trimester placentae from termination of pregnancy due to severe fetal abnormalities<br>Group 3: Placentae from T21-affected pregnancies (matched with Group 2 based on gestational age)<br>Group 4: Term placentae from elective caesarean section from healthy mothers with uncomplicated pregnancies
Maymon, Ron, Jauniaux, Eric, and Moroz, Chaya (2002)	p43-PLF	Trisomy 21 karyotyping	N = 10 (healthy pregnant women undergoing cervical dilatation) and N = 10 (healthy)
Metzenbauer et al. (2002)	Placental volume	Diagnosis of fetal aneuploidy was either performed prenatally or postnatally	N = 2863, with N = 9 (T21), N = 4 (T18), N = 2 (T13), N = 1 (Turner's syndrome), N = 1 (48,XXY + 21)
Thirunavukarasu et al. (2002)	Molecular weight forms of inhibin A (70k vs 30-40k)	Down Syndrome diagnosis	Maternal serum: N = 23 (DS) matched 1:1 using a pool of over 46 healthy samples from varying sources<br>Amniotic fluid: N = 12 (DS) matched 1:1 using a pool of over 57 healthy samples from varying sources<br>Placenta: N = 3 (DS singleton pregnancies) and N = 11 (normal)
Farina et al. (2003)	Cell-free fetal DNA (versus AFP, uE3, hCG, and inhibin A)	Down Syndrome diagnosis	N = 15 case-control sets of Down Syndrome male fetuses
Prusa et al. (2003)	hTERT, LIFR and BMPR II.	Trisomy 21 karyotyping	N = 4 for amniocentesis, N = 4 for placental biopsies

Sutton, Jaime M. and Cole, Laurence A. (2003)	sd-ITA	Trisomy 21 karyotyping	N = 10 (DS) and N = 10 (healthy) urine samples within 16-18 weeks of pregnancy and high ITA concentrations
Frendo et al. (2004)	Abnormally glycosylated hCG	Trisomy 21 karyotyping	Maternal serum: N = 499 (DS), N = 894 (Control)<br>Maternal serum hCG clearance: N = 24 (DS), N = 23 (Control)<br>Placental tissue for trophoblast culture: N = 5 (DS), N = 5 (Control)
Palomaki et al. (2004)	ITA	Trisomy 21 karyotyping	N = 2023 unaffected, N = 28 (DS), and N = 4 pregnancies with other chromosome abnormalities)
Wald et al. (2004)	Maternal serum: PAPP-A, free h-hCG, total hCG, uE3, AFP<br>Maternal urine: ITA, beta-core fragment, total hCG and free beta-hCG	Trisomy 21 karyotyping	N = 101 (DS) with each case matched to 5 normal unaffected pregnancies
Banerjee et al. (2005)	Beta-hCG and LHCGR gene	Trisomy 21 confirmed by biochemical, molecular and cytogenetic analyses	CVS sample: N = 58 (DS) and N = 1060 (Normal)<br>Tissue sample (for RNA analysis): N = 41 (DS)
Palomaki et al. (2005)	ITA	Trisomy 21 karyotyping	Maternal serum samples of N = 54 (DS) were case matched 1:5 with N = 276 (Normal)
Weinans et al. (2005)	ITA		Maternal serum samples of N = 24 (DS) and N = 320 (Normal)
Go et al. (2006)	C21orf105 mRNA	Trisomy 21 karyotyping	Maternal plasma: N = 12 (DS) and N = 28 (normal), the DS samples were matched 1:2 with normal samples
Laigaard et al. (2006)	ADAM 12	Trisomy 21 karyotyping as determined by cytogenetics laboratories, the National Down's Syndrome Cytogenetic Register, and the maternity units inwhich the fetus was delivered	Maternal serum: N = 218 (DS) and N = 389 (gestational age matched normal pregnancies)
Chim et al. (2008)	Differential methylated CpG sites on Chromosome 21, U-PDE9A and U-CGI137	Rapid clearance of marker from the circulation upon delivery of the fetus, indicating prenatal detection of fetal sequences on chromosome 21	U-PDE9: N = 12 maternal plasma third-trimester samples and 24h postpartum<br>U-CGI137: N = 8 maternal plasma third-trimester samples with only 7 out of the 8 with samples available post-delivery
Klugman et al. (2008)	TRAIL and KRT8	Trisomy 21 karyotyping	Placental biopsies: N = 11 (DS) and N = 8 (Control)
Papadopoulou et al. (2008)	hGPH	Trisomy 21 karyotyping	Maternal serum samples N = 21 (DS, second trimester) and N = 62 (healthy controls with birth weight appropriate for gestational age)

Linskens et al. (2009)	free β-hCG and PAPP-A	Trisomy 21 karyotyping	Maternal serum from early trimester pregnant mothers with either monochorionic or dichorionic twins N = 200 (control) and N = 2 (DS, three fetuses in the 2 pregnancies)
Qureshi et al. (2009)	PCNA	Trisomy 21 karyotyping	Placentas: N = 18 (DS, 12 second trimester and 6 third trimester) gestational age matched to N = 10 (controls, 5 second trimester and 5 third trimester)
Du et al. (2011)	CpG sites AIRE, POTE, DSCR4	Rapid clearance of marker from the circulation upon delivery of the fetus, indicating prenatal detection of fetal sequences on chromosome 21	N = 10 normal pregnancies (five from the first trimester (two female fetuses and three male fetuses) and five from the third trimester (three female fetuses and two male fetuses))
Sun et al. (2011)	SOD1, ERp29, HSP27, PRDX6	Trisomy 21 FISH-based karyotyping	Maternal placentas: N = 19 (DS) and N = 17 (Normal)
Zhang et al. (2011)	CpG sites AIRE to RASSF1A ratio	Trisomy 21 karyotyping	N = 4 (DS) and N = 384 (Control)
Chen et al. (2012)	Altered proteins belonging to two major functional groups, cytoskeleton structure and regulation and transport. Previously reported: AAT, α-crystallin, ApoA-I, CTSD, HSPB1, MDH, MBP, PRDX2, PRDX6, SAP, TCP1, TTR, VIM, VDAC2<br>New: ASB17, ANXA2, ANXA5, ATXN3, CALR, CSH1, PPIB, XRCC2, EVPL, LPLA2, BLVRB, GRK4, LGALS1, GBP2, KRT8/18, KRT222, KLR, GLO1, ACP1, MPP1, MSP1, MRPP2, PFN1, P4HB, RCN1, NEK7, SRI, SPRED2, VIPR1.	Trisomy 21 karyotyping	N = 5 (DS) and N = 5 (Control)
Eckmann-Scholz et al. (2012)	27,578 differentially methylated CpG sites from more than 14,000 genes	Trisomy 21 karyotyping	N = 3 (Trisomy 21), N = 6 (Trisomy 18), and N = 54 (Control)
Jin et al. (2013)	TET1, TET2, REST genes	Trisomy 21 karyotyping	Placenta villi samples N = 11 (DS) and N = 6 (Normal)
Lim et al. (2014)	34 miRNAs of placental origin	Trisomy 21 karyotyping	N = 4 (DS) and N = 5 (Normal)
Munnangi et al. (2014)	PAPP-A2	Trisomy 21 karyotyping	Maternal serum: Trisomy 21 pregnancies (N = 30) and normal pregnancies (N = 142)<br><br>Placental tissue: N = 7 for Trisomy 21

Yin et al. (2014)	CGI149, CGI045, HLCS-1, and HLCS-2 DNA methylated sites		Placental tissues: N = 11 (DS) and N = 15 (Normal)
Pinilla et al. (2015)	Cystathionine β-synthase	Trisomy 21 karyotyping	Placental tissues: N = 6 (DS) and N = 16 (Normal)
Lee et al. (2016)	FSMR-E, FSMR-U1, and FSMR-U2	Trisomy 21 karyotyping	Blood samples: N = 2 (Nonpregnant euploid women), N = 5 (pregnant euploid women)<br>Placental samples: N = 4 (pregnant euploid women), N = 5 (DS)
Lim et al. (2016)	DNA methylation patterns of 207 genes on Chromosome 21	Trisomy 21 karyotyping	Placental samples: N = 6 (DS) and N = 7 (Normal)
Svobodová et al. (2016)	754 miRNAs of placental origin	Trisomy 21 karyotyping	CVS samples: N = 16 (DS) and N = 14 (Normal)
Lim et al. (2017)	110 candidate genes in whole genome	Trisomy 21 karyotyping	Placental samples: N = 8 (DS) and N = 10 (Normal)
Lim et al. (2018)	110 candidate genes in whole genome	Trisomy 21 karyotyping	Placental samples: N = 7 (DS) and N = 10 (Normal)
Wong et al. (2018)	APP, ETS2, SOD1, and HMGN1	Trisomy 21 karyotyping	Placental samples: N = 34 (DS) and N = 37 (Normal)
Lim et al. (2019)	DNA methylation of CpG sites distributed across the whole genome	Trisomy 21 karyotyping	CVS samples: N = 5 (DS) and N = 5 (Normal)
Leon-Martinez et al. (2020)	Caspase-2 protein expression	Trisomy 21 karyotyping	Trisomy 21 (n = 9) and euploid (n = 4) age-matched placentas
